# Unilateral Breast Abscess Caused by Escherichia coli in an 18-Year-Old Non-lactating Female: A Case Report

**DOI:** 10.7759/cureus.93451

**Published:** 2025-09-28

**Authors:** Yasser A Abd Alsamad, Abdullah S Alsharif, Bashirat L Giwa, Maryam E Al Ali

**Affiliations:** 1 Department of Clinical Sciences, College of Medicine, University of Sharjah, Sharjah, ARE; 2 Infectious Diseases, University Hospital Sharjah, Sharjah, ARE; 3 General Surgery, Dubai Health Authority, Dubai, ARE

**Keywords:** abscess, breast, case report, e. coli, infection

## Abstract

Breast abscesses are commonly encountered in lactating women, typically due to *Staphylococcus aureus*. By contrast, breast abscess in non-lactating women is rare and tends to be a diagnostic challenge. Uncommon pathogens such as *Salmonella *or *Escherichia coli* (*E. coli*) rarely present in the literature as causes of breast abscess. This report highlights the case of an 18-year-old previously healthy female and non-lactating woman who presented with a painful, red, and swollen right breast. Physical examination and ultrasound investigations confirmed the presence of a breast abscess. Surgical drainage and pus culture were done, and *E. coli* was confirmed as the causative organism. She was successfully treated and discharged with orders to return for dressing changes. This case emphasizes the importance of considering atypical organisms like *E. coli* in non-lactational breast abscesses and tailoring antibiotic therapy based on culture results. Further research is warranted to better understand the underlying pathophysiology and to compare the outcomes of surgical drainage versus antibiotic therapy alone in such uncommon presentations.

## Introduction

Breast abscess is a disease that usually affects lactating, breastfeeding women. It tends to occur following trauma during lactation; however, the incidence in non-lactating women has been documented despite it being far less [1]. In young women, specifically non-lactating, it is vitally important to rule out other etiologies, such as inflammatory carcinoma.

Typically, breast abscesses are due to normal skin flora such as *Staphylococcus aureus* or *Streptococcus* [2,3]. However, our case was due to *Escherichia coli* (*E. coli*) infection, and we also found a somewhat similar case report of *E. coli* breast abscess published in 2024, although it was noted to be recurrent [4]. Sometimes, cases of anaerobic bacterial infection, such as *Salmonella*, causing breast abscesses have been reported [5,6]. Finally, a similar report of bilateral breast abscess due to typhoid fever has been reported; however, the frequency of breast abscesses in young, non-lactating women remains low [7,8].

## Case presentation

An 18-year-old previously healthy female came to the emergency department with a six-day history of pain, swelling, and redness in her right breast. The pain was constant and severe for all six days, but the swelling had initially started off small, according to the patient, and continued increasing in size. She denied any previous history of breast pain, swelling, or trauma. She reported no associated symptoms such as fever, rigors, fatigue, or nipple discharge.

The patient is single, and she's not pregnant, lactating, or sexually active. She did not take any medications routinely and has no history of drug allergies. In addition, she had no previous hospitalizations or surgeries. She denied cigarette or alcohol use and had no recent travel history. Finally, she does not have any chronic illnesses such as diabetes or autoimmune disease.

The review of systems was unremarkable. The patient reported no nasal blockage, headache, or throat pain. There was no chest pain or palpitations. Gastrointestinal symptoms were absent, with normal bowel movements and no abdominal pain, diarrhea, or constipation. Urinary habits were normal, without frequency, urgency, or dysuria. Additionally, there was no limb pain or weakness regarding the musculoskeletal system.

In the emergency department, her vitals were all stable and normal. On examination, the patient was conscious and alert, well appearing, and not in any acute distress.

Breast examination showed a lower-quadrant right breast abscess, approximately 5 x 3 cm, which was tender to palpation. No palpable lymph nodes or other swellings were felt; there was no nipple discharge.

Laboratory investigations revealed a normal white blood cell count of 4.57 ×10^9^/L (normal: 4-10 x 10^9^/L). Hemoglobin was mildly reduced at 10.6 g/dL (normal: 12-16 g/dL), with a hematocrit of 31.1 (normal: 36-46%). Platelet count was within normal limits at 291 ×10^9^/L (normal: 150-400 x 10^9^/L). C-reactive protein (CRP) measured 4 mg/L, falling within the normal range (normal: 0-5 mg/L).

**Figure 1:**
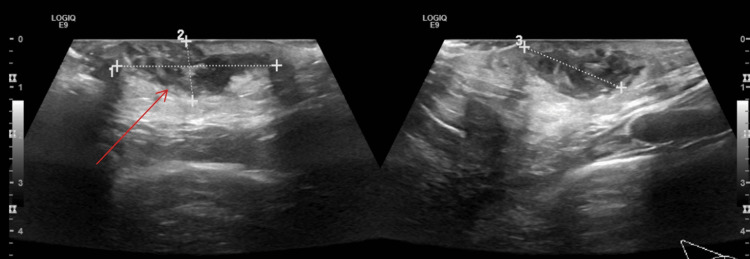
Right breast ultrasonography The arrows point at a large breast abscess measuring 33 x 12 x 22 mm.

A large spiculated collection was seen seated at the subcutaneous plane of the lower inner quadrant (breast root), measuring 33 x 12 x 22 mm, with an approximate volume of about 4.7 ml (Figure 1). It was seen displacing turbid hypoechoic content with echogenic debris. It showed extensive interstitial hyperemia of the subjacent subcutaneous tissue. The picture was indicative of abscess formation. No evident deep extension would be encountered. Reactionary looking sub-centimetric axillary lymph nodes were noted.

A diagnosis of right breast abscess was made, and the patient was promptly initiated on prophylactic ceftriaxone along with analgesics for pain management. She was then kept NPO (nil per os) and prepared for incision and drainage of the breast abscess.

She was operated on under general anesthesia in the supine position. Cleaning and draping were done, an incision was made, and a gush of pus came out, which was sent for culture. All loculi were broken, and the breast abscess was cleaned. Wound packing and pressure dressing were done.

Postoperatively, the patient had mild pain at the wound site, she passed gas and stool, and had no urinary retention. She was then discharged with instructions to return to the outpatient clinic for a dressing change. Vitals were all stable.

Pus culture revealed *E. coli* growth. It was sensitive to amoxicillin/clavulanic acid, cefotaxime, ceftriaxone, ciprofloxacin, piperacillin/tazobactam, and trimethoprim/sulfamethoxazole.

Medications given were Ibuprofen and paracetamol for analgesia, with pantoprazole for gastrointestinal prophylaxis. Ceftriaxone was administered intravenously for three days, and then she was discharged home.

## Discussion

Breast abscesses are rarely seen in non-lactating women. An abscess is a localized collection of pus in any part of the body, and it is usually managed with incision and drainage followed by a course of antibiotics [9]. However, some articles mention that antibiotics could be enough for non-lactational women and incision and drainage are not needed [10], keeping in view that this might be more applicable to smaller abscesses.

Breast abscesses have been well-reported in the literature and are usually due to well-known bacteria such as *Staphylococcus aureus*. In addition, lactation is the most important risk factor for the development of breast abscess [1]. In our patient, she did not have the risk factor of lactation, and the pus culture did not grow *Staphylococcus* or *Streptococcus* species; instead, it grew *E. coli,* which is a bacterium found in the gastrointestinal tract and is a common bacterium in urinary tract infections, which raises the concern of how these bacteria reached the breast.

The pathophysiology of breast abscesses is the blockage and obstruction of ducts, which leads to the formation of abscesses. However, in non-lactating females, the pathophysiology could be due to trauma or chronic diseases such as diabetes [11].

Al-Ishaq et al. described a breast abscess caused by *Salmonella* in a non-lactating female patient, which highlights a trend that shows that non-lactating women can have a wider range of bacterial organisms causing breast abscesses [12].

Palanisamy et al. highlighted a patient with recurrent unilateral breast abscess, which was later found to be due to *E. coli*; this raises a concern that *E. coli* could be associated with non-lactating women, and it is an etiology to be considered when dealing with recurrent breast abscesses [4].

Even rarer bacterial organisms involved in breast abscess causation, such as *Gleimia* and *Mycobacterium,* were also identified in case reports [13,14].

Moreover, the management of breast abscesses starts with needle aspiration, either image-guided or non-image-guided. Aspiration was found to have better outcomes and shorter healing time and does not require the use of general anesthesia. Repeated aspirations may be needed to achieve cure. Incision and drainage are the primary treatments for breast abscesses larger than 5 cm. Regardless of whether aspiration or surgical drainage is performed, antibiotics should be given concurrently [15].

An older systematic review done in 2015, found that there is no difference between needle aspiration and incision and drainage [16].

Therefore, a more recent systematic review done in 2023, had a consensus that said to have needle aspiration as first line due to better advantages and lower complication risks. Cure rate however was not significantly different between needle aspiration and Incision and drainage. Finally, Incision and drainage needs to be done for large volume abscess, multicompartmental abscesses and abscesses that have not responded to needle aspiration [17].

## Conclusions

The literature is conflicted regarding the treatment of breast abscesses in non-lactational women, as some prefer antibiotics alone. Despite that, surgical drainage and proper antibiotics are the preferred management in unilateral breast abscesses, targeting both typical and atypical microbes. Further research will be needed to find out the pathophysiology and mechanism of how *E. coli* can cause breast abscesses.
